# Triptolide Induces Liver Injury by Regulating Macrophage Recruitment and Polarization via the Nrf2 Signaling Pathway

**DOI:** 10.1155/2022/1492239

**Published:** 2022-06-20

**Authors:** Li Liu, Xi Zhang, Xin Xing, Ismail Mohammed, Xiao-ting Xu, Zhen-zhou Jiang, Tao Wang, Xin Huang, Xin-zhi Wang, Lu-yong Zhang, Li-xin Sun

**Affiliations:** ^1^Jiangsu Center for Pharmacodynamics Research and Evaluation, China Pharmaceutical University, Nanjing 210009, China; ^2^Key Laboratory of Drug Quality Control and Pharmacovigilance, China Pharmaceutical University, Nanjing 210009, China; ^3^Center for Drug Screening and Pharmacodynamics Evaluation, School of Pharmacy, Guangdong Pharmaceutical University, Guangzhou 510006, China

## Abstract

Triptolide (TP) has limited usage in clinical practice due to its side effects and toxicity, especially liver injury. Hepatic macrophages, key player of liver innate immunity, were found to be recruited and activated by TP in our previous study. The nuclear factor-erythroid-2-related factor 2 (Nrf2) pathway exerts a protective role in TP-induced liver damage, but its effect on the functions of hepatic macrophage has not been elucidated. Here, we determined whether TP can regulate the recruitment and polarization of hepatic macrophages by inhibiting Nrf2 signaling cascade. Our results demonstrated that TP inhibited the Nrf2 signaling pathway in hepatic macrophages. The changes in hepatic macrophages were responsible for the increased susceptibility toward inflammatory stimuli, and hence, TP pretreatment could induce severe liver damage upon the stimulation of a nontoxic dose of lipopolysaccharides. In addition, the Nrf2 agonist protected macrophages from TP-induced toxicity and Nrf2 deficiency significantly aggravated liver injury by enhancing the recruitment and M1 polarization of hepatic macrophages. This study suggests that Nrf2 pathway-mediated hepatic macrophage polarization plays an essential role in TP-induced liver damage, which can serve as a potential therapeutic target for preventing hepatotoxicity induced by TP.

## 1. Introduction

*Tripterygium wilfordii* Hook. f. (TWHF), a Chinese herbal remedy, has been commonly applied to treat inflammation and autoimmune diseases for hundreds of years [1, 2]. Triptolide (TP) is a diterpene extracted from TWHF in 1972 [3], which possesses excellent pharmacological activities, including proapoptotic, antiproliferative, anti-inflammatory, and immune modulatory activity [4, 5]. However, the clinical use of TP is often limited by its severe toxic effects and narrow therapeutic window. Liver injury is the most common adverse reaction caused by TP, which has attracted widespread concern [6].

A clinical study showed that TWHF preparations containing TP could dramatically increase the level of serum transaminase and induce severe liver injury [7]. However, a high dose of TWHF preparations or TP, which was equivalent to 10–20 times of clinical dose, could slightly elevate the serum levels of aspartate aminotransferase (AST) and alanine aminotransferase (ALT) (up to 2–3 times normal) in mice [8, 9] and the histological evaluation did not show obvious liver injury. Our previous studies showed that the effect of TP differed greatly when the animals were bred in different environments. TP could lead to severe liver injury in conventional environment, where the transaminase level of mice was nearly ten times higher than that in the barrier system [10, 11]. This phenomenon might be related to the promising immunosuppressive activity of TP, and a long-term TP administration could trigger inflammatory responses in the liver. This hypothesis was verified by our previous work [12], in which a nontoxic dose (0.1 mg·kg^−1^) of lipopolysaccharides (LPS) was used to stimulate the immune response of mice. In fact, the pretreatment of TP could induce liver hypersensitivity after LPS stimulation, thus resulting in hepatic apoptosis and necrotic cell death.

Hepatic macrophages play key roles in ensuring rapid responses to liver injury, maintaining tissue homeostasis, and regulating the development and progression of liver diseases [13]. Our previous study found that TP induced the recruitment and activation of macrophages, as well as inhibited their phagocytic function [14]. Based on the abovementioned studies, we assume that the changes in macrophage functions may be associated with the enhanced susceptibility of the liver to inflammatory stimuli. Nuclear factor-erythroid-2-related factor 2 (Nrf2), a protein with a molecular weight of 95–110 kilodalton belonging to the basic leucine zipper transcription factor, is a crucial transcription factor that confers protection towards liver cells via antioxidant and anti-inflammatory mechanisms [15]. The Nrf2 pathway has been found to play a protective role in TP-induced liver damage through its antioxidant properties [16]. However, it remains unclear whether Nrf2 can affect the phenotype and function of macrophages in response to inflammatory stimulation.

To understand the toxicity of TP from a new perspective, we explored the role of hepatic macrophages in mediating the increased susceptibility of liver injury to inflammatory stimulation induced by TP, as well as the effects of the Nrf2 signaling pathway on the expression and function of hepatic macrophages during TP-induced liver injury.

## 2. Materials and Methods

### 2.1. Preparation of TP and LPS

TP (Sanling Biotech, Guilin, China) was dissolved in 1,3-propanediol, kept at −20°C, and diluted with CMC-Na (0.5%) to obtain the desired concentrations. LPS (Sigma-Aldrich, MO, USA) was diluted with phosphate buffer solution (PBS) and filtered using the milli-pore filter (0.22 *μ*m).

### 2.2. Animal Maintenance and Treatment Procedure

SPF-grade C57BL/6 mice (6–8 weeks old) and B6/J-Nrf2em1Cd/Nju mice (6–8 weeks old) were supplied by the Vital River Laboratory Animal Technology (Beijing, China) and Institute of Biomedicine of Nanjing University, respectively. All mice had ad libitum access to water and food. The experimental protocol was approved by the Ethics Committee of China Pharmaceutical University (approval no. 2021-08-005) and was conducted in compliance with the guidelines of the Laboratory Animal Management Committee of Jiangsu Province. The doses and routes of administration of TP (500 *μ*g·kg^−1^, intragastric administration) and LPS (0.1 mg·kg^−1^, intraperitoneal injection) were referred to our previous method [12, 17]. The LPS dose of 0.1 mg·kg^−1^ was demonstrated to be nontoxic in a previously published study [18]. After LPS administration for 4 hours, the blood and liver tissue specimens were collected for subsequent experiments.

### 2.3. Blood Chemical Analysis

After centrifugation (3,000 rpm, 4°C, 10 minutes), the serum samples were subjected to liver function tests using the AST and ALT assay kits (Weiteman Biotech, Nanjing, China).

### 2.4. Histopathological Evaluation

After immersion in 10% formaldehyde solution for 24 hours, the liver tissues were fixed in 70% ethanol, paraffin-embedded, and sectioned at 8–10 *μ*m thickness. Thereafter, the morphology of each tissue section was examined by hematoxylin and eosin (H&E) staining.

### 2.5. Immunofluorescence Analysis

After mounting on a microscope slide and blocking for 1 hour, the fresh liver cryosections were fixed in paraformaldehyde (4%) for 1 hour. Subsequently, the tissue sections were stained with Alexa Fluor® 647 anti-mouse F4/80 at 4°C. On the next day, 4′,6-diamidino-2-phenylindole (DAPI) was employed for nuclei visualization. The fluorescence intensity of each section was examined using a FV1000 microscope (Olympus, Japan).

### 2.6. Isolation of Primary Hepatocytes and Hepatic Macrophages from Mice

The modified two-step perfusion method was used to isolate primary hepatocytes from each mouse [19]. All liver cells were harvested and centrifuged at low speed for separating the hepatocytes from the nonparenchymal cells. The hepatic macrophages were further separated from the nonparenchymal cells using a 25% and 50% Percoll gradient centrifugation technique.

### 2.7. Flow Cytometry

To block Fc receptors, the collected primary hepatic macrophages were incubated with CD16/32. Subsequently, the hepatic macrophages were incubated with specific antibodies, including F4/80, CD45, CD86, and CD11b. The intracellular marker CD206 was employed to break the membrane. ROS levels were determined by DCFH-DA (Beyotime Biotechnology, Shanghai, China). All samples were subjected to flow cytometry (Becton Dickinson, CA, USA), and data analysis was conducted with FlowJo v10.0 software (FlowJo, OR, USA).

### 2.8. Cell Culture and Viability Assays

The Raw264.7 cell line was supplied by the China Cell Culture Center (Shanghai, China). The cells were cultured in DMEM containing 10% fetal bovine serum (FBS) (Gibco, NY, USA) and maintained at 37°C and 5% CO2.

Cell viability was determined using the CCK8 assay kit (Donjindo Laboratories, Kumamoto, Japan). The cells (2 million cells/well) were grown in a 96-well plate at 37°C for 24 hours and then exposed to different concentrations of TP. Afterwards, 10 *μ*L of CCK8 was added into each well, followed by an incubation period of 1–4 hours. The absorbance values were measured at 450 nm.

### 2.9. RNA Isolation, Reverse Transcription, and Real-Time Quantitative PCR (RT-qPCR)

Total RNA was extracted from the liver, hepatic macrophages, and Raw264.7 cells using the TRIzol reagent (Vazyme Biotech, Nanjing, China). The concentration and purity of RNA samples were evaluated using a NanoDrop 2000 spectrophotometer (Thermo Fisher, MA, USA). cDNA synthesis was conducted with HiScript® Q RT SuperMix for qPCR (Vazyme Biotech) by following the kit's protocol. The IQ™ 5 RT-PCR detection system (Bio-Rad Laboratories, CA, USA) was used to detect the mRNA expression of target genes. HPRT1 was used as a housekeeping gene. The sequences of each primer pair are presented in Supplementary Table 2.

### 2.10. Western Blotting

Total protein was isolated from the mouse liver and Raw264.7 cells using the RIPA buffer (Beyotime Biotechnology). The total protein content was determined using the BCA protein assay kit and then mixed with loading buffer (Bio-Rad Laboratories). After separation through SDS-PAGE, the protein samples were transferred onto PVDF membranes and the blocking solution containing 5% BSA was added for 1 hour. Later, the membranes were incubated overnight with primary antibodies at 4°C and then with HRP-conjugated polyclonal secondary antibodies. Lastly, the protein blots were examined using an ECL detection kit (Millipore, MA, USA).

### 2.11. Statistical Analysis

Statistical tests were conducted with GraphPad Prism v8.0 (GraphPad Software Inc., CA, USA). All values were shown as mean ± SEM. The means between two groups were compared using the unpaired Student's *t* − test. Other data were evaluated with one-way or two-way ANOVA followed by Tukey's multiple comparison test. A *P* value of <0.05 was deemed statistically significant.

## 3. Results

### 3.1. TP Affects Nrf2 Signal Transduction Both In Vitro and In Vivo

**Figure 1:**
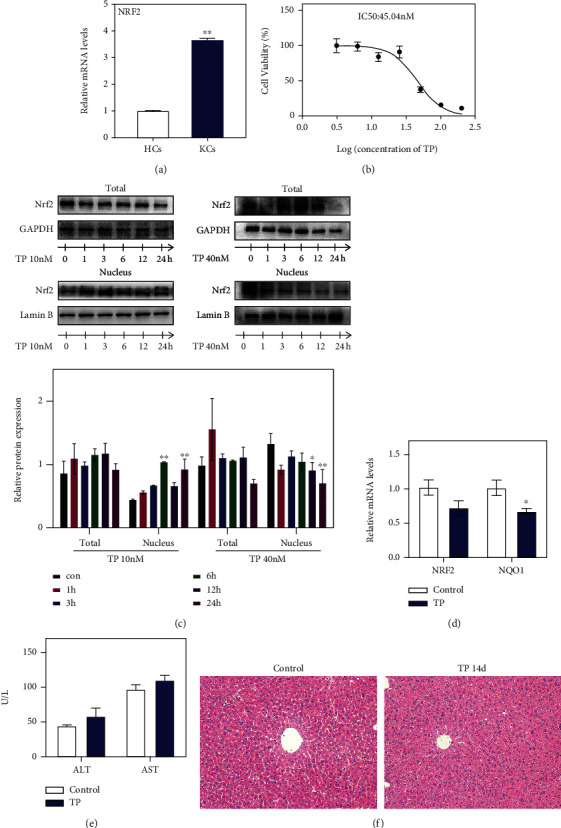
Effects of TP on the Nrf2 signal pathway both in vivo and in vitro. (a) Comparison of Nrf2 mRNA expression in primary hepatocytes (HCs) and Kupffer cells (KCs) (*n* = 5). (b) Changes on the viability of Raw264.7 cells administrated with different concentrations of TP for 24 hours (*n* = 6). (c) Effect of TP on the transportation of Nrf2 to the nucleus in Raw264.7 cells (*n* = 3). (d) Changes of Nrf2 and NQO1 in the liver of mice after TP treatment for 14 days (*n* ≥ 5). (e) Changes in the serum ALT and AST in female and male mice administrated with TP for 14 days (*n* = 6). (f) Representative images of HE-stained livers from female and male mice; inflammatory infiltration (black arrows), 200x magnification. Data are presented as mean ± SEM, ^∗^*P* < 0.05 and ^∗∗^*P* < 0.01 vs. control.

Nrf2 responds to environmental stressors by activating numerous cytoprotective, antioxidant, and detoxifying enzymes [20]. Nrf2 deficiency exaggerates oxidative stress, dysregulates autophagy, and induces cytotoxicity [21]. To compare the expression levels of Nrf2 in different types of liver cells, we isolated primary hepatocytes and hepatic macrophages from each mouse. Compared with hepatocytes, the level of Nrf2 was highly expressed in hepatic macrophages (Figure 1(a)). Recent studies indicate that macrophage Nrf2 mediates innate proinflammatory responses [22] and changes in the macrophage phenotype [23]. To uncover whether the Nrf2 pathway can regulate the function of hepatic macrophages, the effects of TP on Nrf2 expression were detected both in vitro and in vivo. First, Raw264.7 cells were employed to determine the effect of TP on Nrf2 pathway activation in vitro. The viability of Raw264.7 cells was inhibited by TP in a dose-dependent fashion, and the IC50 is 45.04 nM (Figure 1(b)). According to the IC50 value, the doses of 10 and 40 nM were chosen for further experiments. The translation of Nrf2 to the nucleus was promoted by low doses of TP, while high concentrations of TP inhibited the nuclear translation of Nrf2 at all time points (Figure 1(c)). Consistent with the in vitro results, there was a decrease in the mRNA expression of Nrf2 and NQO1 (a downstream gene of Nrf2) after TP treatment (Figure 1(d)). Although TP inhibited Nrf2 signal transduction in the liver, it did not directly cause liver damage (Figures 1(e) and 1(f)).

### 3.2. Nrf2 Agonist Protects Macrophages from TP-Induced Toxicity and Inhibits M1 Polarization

**Figure 2:**
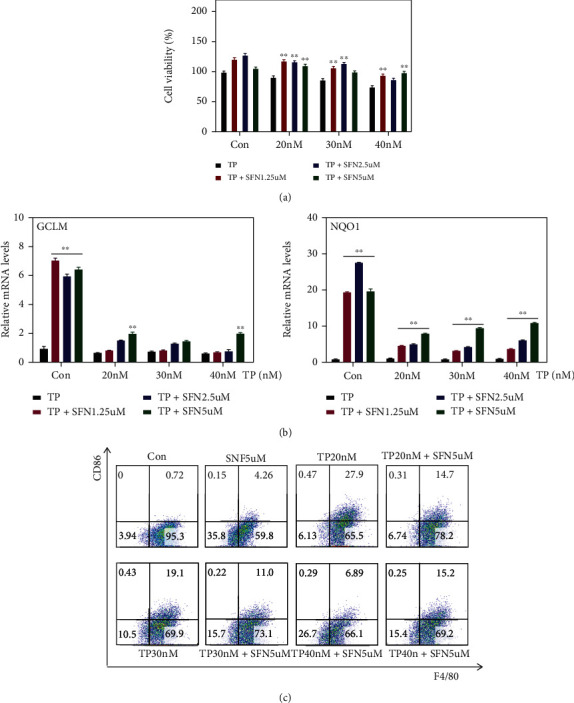
Nrf2 agonist protects macrophages from TP-induced toxicity and inhibits M1 polarization. (a) Effect of SFN on Raw264.7 cell viability after TP treatment (*n* = 6). (b) Effect of SFN on the Nrf2 signaling pathway in Raw264.7 cells (*n* = 6). (c) Changes in M1 macrophages in Raw264.7 cells after SFN pretreatment. Data are presented as mean ± SEM, ^∗^*P* < 0.05 and ^∗∗^*P* < 0.01 vs. TP.

The Nrf2 signaling pathway has been reported to play an antioxidant role in nonalcoholic steatohepatitis [24], liver fibrosis [25], and liver ischemia/reperfusion [26], which may offer a novel potential strategy for the prevention and treatment of liver diseases [27]. Sulforaphane (SFN), an agonist of Nrf2 that has been demonstrated to promote the nuclear translocation of Nrf2 and suppress oxidative stress [28], was chosen to verify the protective role of the Nrf2 pathway in TP-induced cytotoxicity. Pretreatment with SFN for 2 hours could activate the downstream genes of Nrf2 (Figure 2(a)) and restore the viability of Raw264.7 cells attenuated by TP (Figure 2(b)). Moreover, SFN also inhibited M1 polarization induced by TP (Figure 2(c)). This indicates that the Nrf2 signaling pathway can mediate the effect of TP on hepatic macrophages.

### 3.3. Nrf2 Deficiency Promotes M1 Polarization and Inhibits M2 Polarization in Hepatic Macrophages

**Figure 3:**
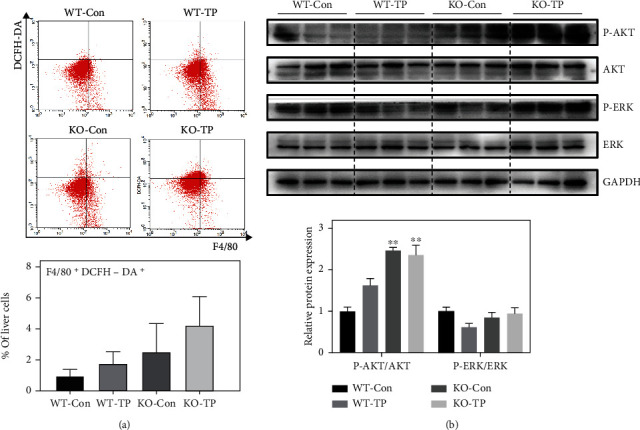
Effects of TP on the ROS pathway in Nrf2^−/−^ mice. (a) The proportion of F4/80^+^DCFH-DA^+^macrophages in the liver (*n* = 5). (b) Changes in the protein expression of ROS-related pathways in the liver (*n* = 3). Data are presented as the mean ± SEM, ^∗^*P* < 0.05 vs. control, ^∗∗^*P* < 0.01 vs. WT-Con.

**Figure 4:**
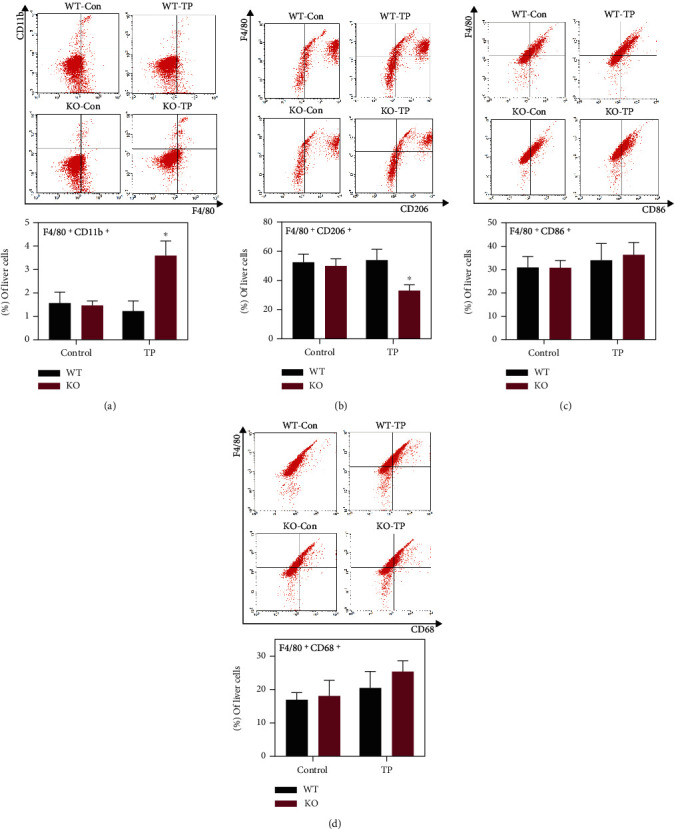
Effects of TP on macrophages in Nrf2^−/−^ mice. (a) Changes in the proportion of monocyte-derived macrophages (F4/80^+^CD11b^+^) in WT and Nrf2^−/−^ mice administrated with TP for 14 days (*n* = 5). (b) Changes in the proportion of M2 macrophages (F4/80^+^CD206^+^) in WT and Nrf2^−/−^ mice administrated with TP for 14 days (*n* = 5). (c) Changes in the proportion of M1 macrophages (F4/80^+^CD86^+^) in WT and Nrf2^−/−^ mice administrated with TP for 14 days (*n* = 5). (d) Changes in the proportion of activated macrophages (F4/80^+^CD68^+^) in WT and Nrf2^−/−^ mice administrated with TP for 14 days (*n* = 5). Data are presented as mean ± SEM, ^∗^*P* < 0.05 vs. WT-TP.

The abovementioned results demonstrated that dysregulation of the Nrf2 pathway was related to the regulatory effect of TP on macrophage functions. Nrf2^−/−^ mice were treated with TP for 14 days to evaluate the important role of Nrf2 in regulating the phenotype and function of hepatic macrophages during TP-induced liver injury. The results showed that Nrf2 deficiency activated ROS and its downstream signaling pathway in the liver after TP administration (Figure 3). TP induced the recruitment of F4/80^+^CD11b^+^ monocyte-derived macrophages (MoMFs) and inhibited the polarization of F4/80^+^CD206^+^ M2 macrophages in Nrf2^−/−^ mice (Figures 4(a) and 4(b)). However, it had no significant effect on M1 polarization, which was revealed by F4/80^+^CD86^+^ expression (Figure 4(c)). CD68 is an inflammation marker of macrophages, and F4/80^+^CD68^+^ cells are known as the activated macrophages. TP slightly increased the proportion of activated F4/80^+^CD68^+^ macrophages, but the difference was not statistically significant (Figure 4(d)).

**Figure 5:**
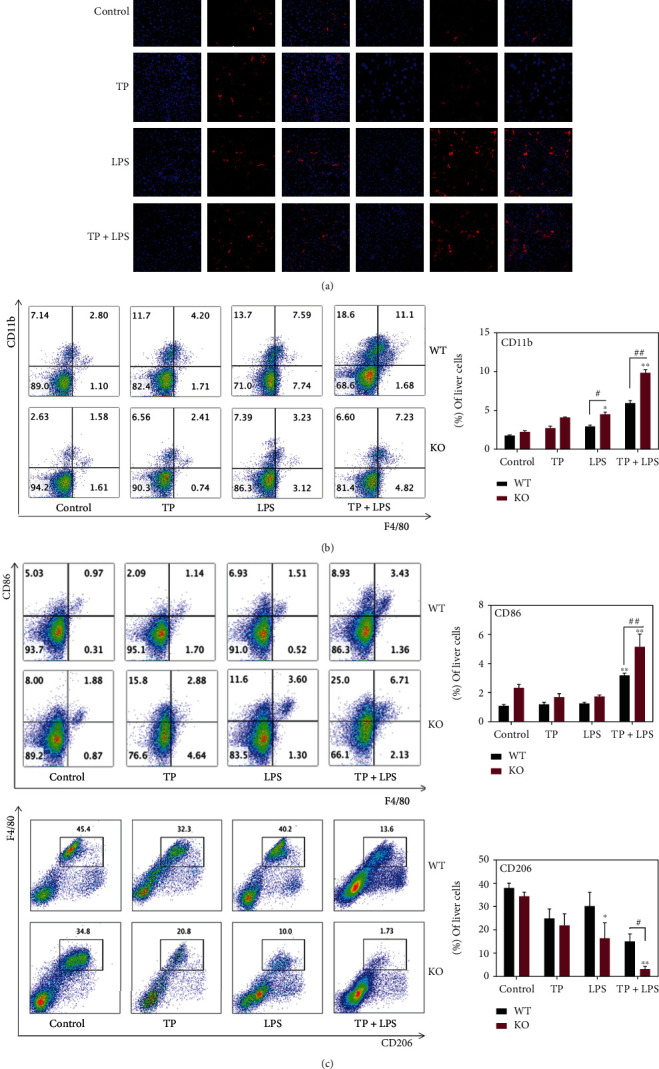
Nrf2 deficiency further promotes M1 polarization in LPS-stimulated mice after TP pretreatment. (a) F4/80 Immunofluorescent staining of the liver. Data are representative of experiments (DAPI: nucleus, F4/80: macrophages). (b) Changes in the proportion of monocyte-derived macrophages (F4/80^+^CD11b^+^) in Nrf2^−/−^ mice administrated with TP and LPS (*n* = 5). (c) Changes in the proportion of liver M1 (F4/80^+^CD86^+^) and M2 (F4/80^+^CD206^+^) macrophages in Nrf2^−/−^ mice administrated with TP and LPS (*n* = 5). Data are presented as mean ± SEM, ^∗^*P* < 0.05 and ^∗∗^*P* < 0.01 vs. WT-control.

It has been proven that TP can inhibit the phagocytic function of primary hepatic macrophages and Raw264.7 cells in a dose-dependent fashion [14]. Thus, we assessed the changes in the function of macrophages leading to the incapable clearance of endotoxins by the liver, which might be associated with an increased susceptibility to inflammatory stimuli. A nontoxic dose of LPS was used as the inflammatory stimulus. The mice were pretreated with 500 *μ*g·kg^−1^·d^−1^ TP for 7 days and injected intraperitoneally with a single dose of 0.1 mg·kg^−1^ LPS 2 hours later on day 7. Interestingly, the number of hepatic macrophages was increased by LPS stimulation after TP treatment (Figure 5(a)), which could be attributed to the recruitment of MoMFs (Figure 5(b)). At the same time, TP pretreatment further promoted the M1 polarization of hepatic macrophages stimulated by LPS (Figure 5(c)). What is more, compared with WT mice, LPS could dramatically increase the proportions of MoMFs and M1-phenotype macrophages in Nrf2^−/−^ mice after TP pretreatment (Figure 5(a)–5(c)).

Hence, we confirmed that TP increased the number of hepatic macrophages, promoted M1 macrophage polarization upon LPS stimulation, and ultimately disrupted the hepatic immune homeostasis via regulating the Nrf2 signaling pathway.

### 3.4. Nrf2 Deficiency Increases the Susceptibility to Inflammatory Stimuli and Causes Severe Liver Damage

**Figure 6:**
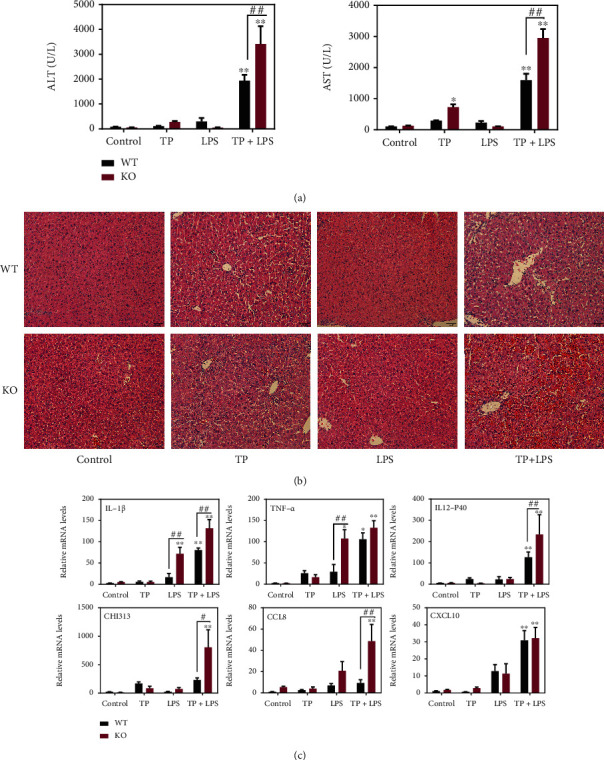
Nrf2 deficiency aggravates the liver injury induced by LPS stimulation after TP pretreatment. (a) Changes in the levels of ALT and AST in WT and Nrf2^−/−^ mice treated with TP and LPS (*n* = 6). (b) Representative images of HE-stained liver from WT and Nrf2^−/−^ mice treated with TP and LPS, 200x magnification. (c) RT-PCR analysis of hepatic M1 genes in WT and Nrf2^−/−^ mice administrated with TP and LPS (*n* = 6). Data are presented as mean ± SEM, ^∗^*P* < 0.05 and ^∗∗^*P* < 0.01 vs. WT-control.

Macrophage polarization is typically viewed as the body's protective response to local inflammation or tissue repair [29]. The M1 polarization of hepatic macrophages in Nrf2^−/−^ mice indicated the imbalance of hepatic immune homeostasis and the occurrence of liver inflammation. To verify whether Nrf2 can induce the susceptibility to inflammatory stimuli and eventually leads to liver damage, the liver functions, histological changes, and proinflammatory cytokine expression were detected in Nrf2^−/−^ mice stimulated by LPS after TP pretreatment for 7 days. Compared with TP or LPS treatment alone, the levels of ALT and AST were markedly increased (*P* < 0.01) by LPS stimulation after 7 days of TP administration (Figure 6(a)). TP or LPS treatment alone did not cause liver damage, but pretreatment with TP increased the susceptibility to LPS stimulation, as manifested by obvious hepatocyte necrosis, nuclear shrinkage, and inflammatory cell infiltration (Figure 6(b)). Furthermore, Nrf2 deficiency caused an exacerbation of liver damage (Figures 6(a) and 6(b)). Compared with the LPS stimulation alone group, the levels of inflammatory cytokines (e.g., TNF-*α*, IL-1*β*, and IL-12) and chemokines (e.g., CHI3l3, CCL8, and CXCL10) were remarkably increased (*P* < 0.01) in Nrf2^−/−^ mice stimulated by LPS after TP pretreatment (Figure 6(c)), which could serve as the main source of M1 macrophages.

Deficiency of Nrf2 aggravated TP-induced susceptibility to inflammatory stimuli by promoting the release of M1 macrophage-related inflammatory factors and chemokines. Our data revealed that the changes in hepatic macrophages regulated by the Nrf2 pathway were involved in the progression of TP-induced liver damage.

## 4. Discussion

TWHF is one of the commonly used immunosuppressants in the clinic, which has a unique curative effect on autoimmune diseases, such as systemic lupus erythematosus and nephrotic syndrome [30]. TP is one of the most common biologically active ingredients found in TWFH, which exhibits almost all the therapeutic activities of TWHF extracts as well as high toxicities. Due to their severe systemic toxicity, TP and its analogs have yet been approved as candidate drugs to date [31]. As an immunosuppressant with good curative effect, TP inevitably affects the liver immune function when exerting its therapeutic effect. In recent years, great attention has been paid to Th17/Treg imbalance mediated by TP [8, 32] but there are relatively few studies on liver immune function, especially the impact on Kupffer cells (KCs). It will help to achieve a new breakthrough in understanding the toxicity mechanism underlying the relationship between hepatotoxicity and immunomodulatory effects of TP. In our previous work, we found that TP induced the recruitment and activation of hepatic macrophages and inhibited their phagocytic function [14]. However, the molecular basis of macrophage recruitment and activation remains obscure. Our results demonstrated that TP inhibited the Nrf2 signaling pathway in hepatic macrophages, thereby promoting the LPS-induced expression of M1 proinflammatory cytokine genes, including TNF-*α* and IL-1*β*. Nrf2 deficiency significantly aggravated the hepatotoxicity of TP via enhancing the recruitment and M1 polarization of hepatic macrophages.

Hepatic macrophages, the most abundant liver immune cells, play critical roles in maintaining liver homeostasis and mediating the pathogenesis of liver injury and repair [33, 34]. Hepatic macrophages consist of liver-resident KCs and MoMFs recruited from the peripheral blood. KCs maintain liver homeostasis through the clearance of pathogenic microbes and phagocytosis of cellular debris. MoMFs infiltrate into the liver tissues when cellular damage occurs and have potent cytokine-producing capacities [35, 36]. Macrophages undergo polarized activation to M1- or M2-like phenotypic states by adapting to the local microenvironment during the progression of liver injury. M1 macrophages have proinflammatory effects on liver injury and inflammation, while M2 macrophages exhibit profibrotic or anti-inflammatory effects on tissue repair [37]. Based on their heterogeneity and functions, hepatic macrophages are able to eliminate pathogenic microbes and promote or inhibit liver inflammation by producing a wide variety of anti- or proinflammatory cytokines and growth factors [38]. Therefore, in this study, we assessed the origin, polarization, and function of hepatic macrophage after pretreatment with TP. Through pathological analyses, we found that TP-induced liver injury in mice was extremely complicated. There was no obvious hepatotoxicity after continuous TP administration, but TP could inhibit Nrf2 signal transduction in the liver and promote M1 polarization of macrophages, indicating the possible role of TP in disrupting liver immune homeostasis.

Compared with the mice with mild liver damage in the barrier environment, the mice in the conventional environment showed remarkable liver toxicity and even death after administration with the same dose of TP for 28 days [10]. Considering the effects of TP on the functions of hepatic macrophages as described above, we speculate that TP-induced hepatotoxicity in the conventional environment is related to an increased susceptibility to inflammatory stimuli, which may be used by the dysfunction of hepatic macrophages and subsequent suppression of the immune system. Our findings demonstrated that preadministration of TP for 7 days could increase the susceptibility of liver injury to LPS stimulation. TP influenced the liver microenvironment by recruiting MoMFs on the one hand and regulated the proinflammatory factors secreted by M1 macrophages on the other. This may explain the way that TP inhibits the function of macrophages, thus promoting the susceptibility to inflammatory stimuli.

Nrf2 is an essential transcription factor that acts by protecting cells from oxidative damage, maintaining redox homeostasis, and regulating anti-inflammatory responses [39]. It has been reported that Nrf2 activation could suppress the levels of proinflammatory cytokines in macrophages independent of the antioxidant mechanism [40, 41] and exert a critical protective effect on TP-induced liver damage [29]. TWHF preparations are used as an immunosuppressant clinically, and it is well recognized that immunity imbalance is involved in TP-induced liver damage [8]. However, previous research on the role of Nrf2 in TP-induced liver damage has only focused on antioxidant responses or apoptosis in hepatocytes [29]. The correlation between Nrf2 and immune modulation, especially the regulation of hepatic macrophages, is neglected. Therefore, we focused on effect of the Nrf2 signaling pathway on the changes of hepatic macrophage phenotypic states, with the aim of identifying potential targets for the treatment of liver damage caused by TP. In this work, we demonstrated that TP (40 nM) inhibited the transportation of Nrf2 protein to the nucleus in Raw264.7 cells and the agonist of Nrf2 restored TP-attenuated cell viability. Furthermore, our results showed that Nrf2 deficiency aggravated the susceptibility of liver injury to inflammatory stimuli by recruiting MoMFs, promoting M1 polarization, inhibiting M2 polarization, and upregulating M1 macrophage-related proinflammatory cytokines.

This study explored the mechanisms of TP-induced liver injury from the perspective of immunosuppression and determined the inhibition of the Nrf2 signaling pathway in hepatic macrophages.

## 5. Conclusions

Taken altogether, our study provides evidence for the effect of TP on the phenotype and function of hepatic macrophages via activation of the Nrf2 pathway, reveals the molecular mechanism of TP-induced liver injury from a new perspective, and identifies the targets for preventing the clinical hepatotoxicity of TWHF preparations.

## Data Availability

The data generated for this study are all included in the manuscript.

## Supplementary Materials

Supplementary Materials Information of the antibodies is shown in Supplementary Table 1. The sequences of each primer pair are presented in Supplementary Table 2.
