# Supplementary material for: Triptolide Induces Liver Injury by Regulating Macrophage Recruitment and Polarization via the Nrf2 Signaling Pathway
Source: Oxid Med Cell Longev. 2022 Jun 20;2022:1492239. doi: 10.1155/2022/1492239 (PMC9236772; doi:10.1155/2022/1492239)
Supplement: Supplementary Materials — Information of the antibodies is shown in Supplementary Table 1. The sequences of each primer pair are presented in Supplementary Table 2. [file 1492239.f1.pdf]

Table 1 Primer sequences

Gene	Primer	Sequence (5' to 3')
HPRT1	Forward	CAG ACT TTG TTG GAT TTG AAA
	Reserve	GCT CAT CTT AGG CTT TGT AT
F4/80	Forward	CTT TGG CTA TGG GCT TCC AGT C
	Reserve	GGC AAG GAG GAC AGA GTT TAT CGT G
CD68	Forward	GCC CGA GTA CAG TCT ACC TGG
	Reserve	AGA GAT GAA TTC TGC GCC AT
CD11b	Forward	CGG TAG CAT CAA CAA CAT
	Reserve	GCA TCA AAG AGA ACA AGG T
IL-1β	Forward	AAT CTC ACA GCA GCA CAT C
	Reserve	AGC AGG TTA TCA TCA TCA TCC
TNF-α	Forward	GCC TCC CTC TCA TCA GTT C
	Reserve	ACT TGG TGG TTT GCT ACG
MCP-1	Forward	TCC ACA ACC ACC TCA AGC ACT TC
	Reserve	GGC ATC ACA GTC CGA GTC ACA C
IL-12p40	Forward	GGA AGC ACG GCA GCA GAA TA
	Reserve	AAC TTG AGG GAG AAG TAG GAA TGG
CCL17	Forward	AGT GCT GCC TGG ATT ACT TCA AAG
	Reserve	CTG GAC AGT CAG AAA CAC GAT GG
CCL22	Forward	TAA CAT CAT GGC TAC CCT GCG
	Reserve	TGT CTT CCA CAT TGG CAC CA
TGF-β1	Forward	ACC AAG GAG ACG GAA TAC
	Reserve	TGT GGA GCT GAA GCA ATA
CD86	Forward	ACG TAT TGG AAG GAG ATT ACA GCT
	Reserve	TCT GTC AGC GTT ACT ATC CCG C
iNOS	Forward	GCC ACC AAC AAT GGC AAC A
	Reserve	CGT ACC GGA TGA GCT GTG AAT T
IL-6	Forward	TCT GAA GGA CTC TGG CTT TG
	Reserve	GAT GGA TGC TAC CAA ACT GGA
CD206	Forward	GGC TTC CGT CAC CCT GTA TGC
	Reserve	ACC TTC CAT CTG CTC CAC AAT CC
Nrf2	Forward	ATATCCCCAGCCACGCTGAAA
	Reserve	CTTGCTCCATGTCCTGCTCTAT
NQO1	Forward	GCTGCAGACCTGGTGATATT
	Reserve	ACTCTCTCAAACCAGCCTTT
CCL24	Forward	GCA GCA TCT GTC CCA AGG
	Reserve	GCA GCT TGG GGT CAG TAC A
FIZZL	Forward	CCA ATC CAG CTA ACT ATC CCT CC
	Reserve	CCA GTC AAC GAG TAA GCA CAG
CHI313	Forward	TCA CTT ACA CAC ATG AGC AAG AC
	Reserve	CGG TTC TGA GGA GTA GAG ACC A

Table 2 Information of antibodies

Antibody	Information
APC anti-mouse CD45 Antibody	BioLegend, 103112
PE anti-mouse F4/80	BioLegend, 123110
Alexa Fluor® 647 anti-mouse F4/80	BioLegend, 123140
FITC anti-mouse/human CD11b	BioLegend, 101206
PerCP/Cy5.5 anti-mouse CD68 Antibody	BioLegend, 137010
PerCP/Cy5.5 anti-mouse CD86 Antibody	BioLegend, 105028
PerCP/Cy5.5 anti-mouse CD206 Antibody	BioLegend, 141716
Antibody against Phospho-p44/42 MAPK (Erk1/2) (Thr202/Tyr204)	Cell Signaling Technology, 4370
Antibody against p44/42 MAPK (Erk1/2)	Cell Signaling Technology , 4695
Antibody against Phospho-Akt (Ser473)	Cell Signaling Technology , 4060
Antibody against NRF2	Proteintech, 16396-1-AP
Antibody against Lamin B	Santa Cruz Biotechnology, sc-6216
Antibody against GAPDH	Santa Cruz Biotechnology, sc-365062
